# A Gelatin-sulfonated Silk Composite Scaffold based on 3D Printing Technology Enhances Skin Regeneration by Stimulating Epidermal Growth and Dermal Neovascularization

**DOI:** 10.1038/s41598-017-04149-y

**Published:** 2017-06-27

**Authors:** Si Xiong, Xianzhu Zhang, Ping Lu, Yan Wu, Quan Wang, Heng Sun, Boon Chin Heng, Varitsara Bunpetch, Shufang Zhang, Hongwei Ouyang

**Affiliations:** 1 Center for Stem Cell and Tissue Engineering, School of Medicine, Zhejiang University, Hangzhou, China; 2 Zhejiang Provincial Key Laboratory of Tissue Engineering and Regenerative Medicine, Hangzhou, China; 3 University of Hong Kong, Faculty of Dentistry, Pokfulam, Hong Kong SAR China; 4 State Key Laboratory for Diagnosis and Treatment of Infectious Diseases, Collaborative Innovation Center for Diagnosis and Treatment of Infectious Diseases, The First Affiliated Hospital, College of Medicine, Zhejiang University, Hangzhou, China; 5 Department of Sports Medicine, School of Medicine, Zhejiang university, Hangzhou, China; 6 Dr. Li Dak Sum & Yip Yio Chin Center for Stem Cell and Regenerative Medicine, School of Medicine, Zhejiang University, Hangzhou, China

## Abstract

One of the key problems hindering skin repair is the deficiency of dermal vascularization and difficulty of epidermis regeneration, which makes it challenging to fabricate scaffolds that can biologically fulfill the requirements for skin regeneration. To overcome this problem, three-dimensional printing was used to fabricate a gelatin-sulfonated silk composite scaffold that was incorporated with basic fibroblast growth factor 2 (FGF-2) through binding with a sulfonic acid group (SO_3_) (3DG-SF-SO_3_-FGF). The efficacy and mechanism by which the 3DG-SF-SO_3_-FGF scaffolds promote skin regeneration were investigated both within *in vitro* cell culture and *in vivo* with a full-thickness skin defect model. The histological results showed that the gelatin-sulfonated silk composite scaffolds promoted granulation, and that incorporation of FGF-2 significantly enhanced the regeneration of skin-like tissues after implantation in rat skin defects for 14 and 28 days. Further investigations demonstrated that 3DG-SF-SO_3_-FGF scaffolds might stimulate dermal vascularization. These findings thus suggest that incorporation of FGF-2 into the 3D printed scaffolds is a viable strategy for enhancing skin regeneration.

## Introduction

Skin is the largest organ in our body and primarily serves as a protective barrier between the body and the external environment. Skin injury is commonly encountered in the clinic due to unavoidable direct exposure of skin to potentially harmful microbial, mechanical, thermal and chemical insults in daily life, as well as internal factors arising from various medical conditions such as diabetes, obesity and aging^1^. Although most cutaneous defects can heal naturally, some acute and chronic skin loss conditions such as venous ulcer and diabetes require hospitalization, resulting in reduced quality of life, as well as substantial socio-economic burden^2^. Approximately 67 million people (1% to 5% of the world’s population) worldwide suffer from chronic skin wounds, with 6.5 million people in the United States alone, resulting in healthcare costs exceeding $25 billion^3^.

The development of skin tissue engineering, which aims to achieve full recovery of skin biological functions, can address pertinent clinical challenges of donor site morbidity and immune-rejection faced in utilizing autografts and allografts in conventional treatment of skin injuries^2^, respectively. Extraordinary advances in skin regeneration have been achieved recently due to rapid progress in the various elements of tissue engineering (seeding cells, scaffolds and growth factors) and their combined synergistic contributions to the field of skin tissue engineering. Nevertheless, the treatment of full thickness skin defects still remains a formidable clinical challenge. Scaffolds seeded with autologous cells, particularly adult stem cells, offer much promise for treatment of skin injuries. However, utilizing autologous cells in skin regeneration is expensive and time-consuming, which would necessitate the development of new strategies^4^. A plausible alternative strategy to utilize autologous cells could be the mobilization and homing of endogenous cells in wound healing through the use of cell-free materials incorporated with biologically active molecules^5–7^.

Three-dimensional (3D) printing is a flexible automated on-demand platform for the free-form fabrication of complex and intricate architectures, enabling the creation of novel bio-scaffolds and viable tissues and organs for regenerative medicine^8^. Different types of biomaterials have been employed to fabricate skin substitutes including naturally derived polymers (alginate, gelatin, collagen, chitosan, fibrin and hyaluronic acid), synthetic molecules (polyethylene glycol, PEG) and their cross-linking agents^9, 10^. Amongst these materials, gelatin, a form of hydrolyzed collagen, has attracted much attention due to its similarity to human extracellular matrix (ECM) and its amenability for suspending cells within a gel environment at low temperature^11^. However, the mechanical weakness and rapid degradation property behavior of gelatin hydrogels are two major disadvantages that severely limit their clinical applications. Therefore, various chemical modifications have been carried out to improve the physical and biological properties of gelatin hydrogels^12, 13^.

Silk fibroin (SF) is a natural polymer produced by a variety of insects and spiders. It has been increasingly investigated and employed as scaffolds to modulate cell behavior. Due to its good biocompatibility, impressive mechanical properties, environmental stability, and non-inflammatory response, SF scaffolds could be utilized as an effective tool in repairing skin, bone, cartilage, ligament and tendon, vascular, neural, tracheal and bladder tissues^14–20^. In the field of drug delivery, SF has also been applied and demonstrated its controlled release kenetics^21–25^. Nevertheless, the slow degradation rate of SF poses a major concern^26, 27^. Recently, there are increasing evidence to suggest that SF degradation can be accelerated by diminishing the formation of beta-amyloid structures or through incorporation of other rapid-degrading materials^28, 29^. Hence, gelatin was incorporated during the initial process of scaffold fabrication. Furthermore, we utilized the 3D printed gelatin grid to provide a base for final molding of the silk fibroin solution, as well as to provide larger pores for speeding up the degradation process.

In this study, we fabricated the 3D-printed scaffold in the form of a “porous grid scaffold”, in which the 3D-printed gelatin grid functioned as the basic structural network that was coated with the sulfonated silk fibroin derivative. This composite scaffold can serve as a “porous magnet” to sequester and concentrate basic fibroblast growth factor (FGF-2). We hypothesize that the 3D-printed gelatin-silk fibroin composite scaffold incorporated with FGF-2 can promote the formation of granulation tissue, which could thus further enhance the repair of full-thickness skin defects.

## Results

### Scaffold characterization

**Figure 1:**
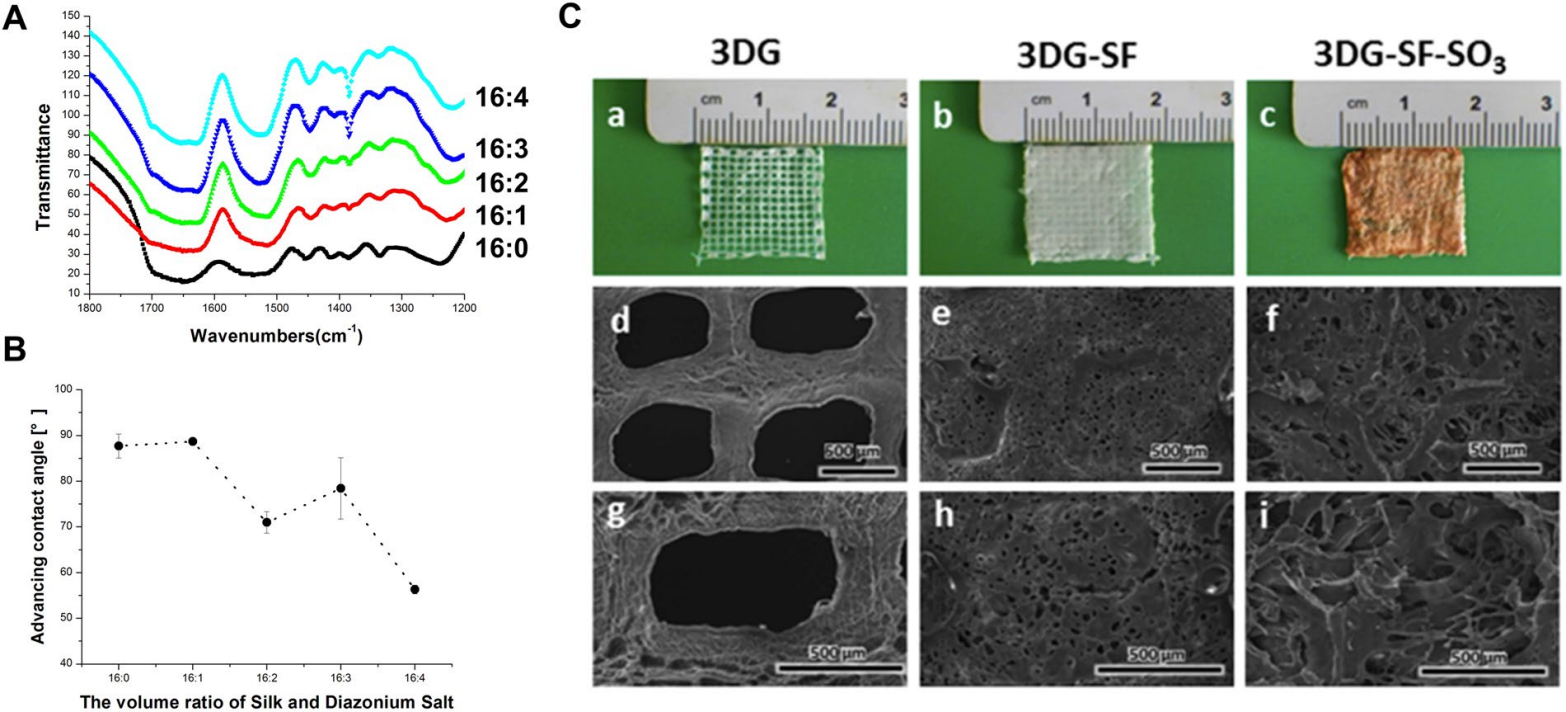
Characterization of scaffolds. (**A**) Comparison of the ATR-FTIR spectra of unmodified and sulfonated silk. (**B**) Advancing contact angles of native SF and SF derivatives with 0, 1, 2, 3 and 4 volumes per 16 volumes of silk. (**C**) Micrographs of 3D printed scaffolds (3DG) (a), SF coated 3D printed scaffold (3DG-SF) (b) and SF derivatives coated 3D printed scaffold (3DG-SF-SO_3_) (c). Scanning electron microscopy (SEM) images of 3DG scaffold (d and g), 3DG-SF scaffold (e and h), and 3DG-SF-SO_3_ scaffold (f and i), with (g,h,i) at high magnification. The thickness of each layer is 100 μm, and the whole thickness of printed gelatin and gelatin coated with sulfonated silk fibroin scaffold is 1 mm. Scale bars, 500 μm.

To stabilize the incorporated FGF-2 within the composite scaffold, diazonium coupling chemistry was used to attach sulfonic acid moieties to tyrosine residues in the native SF. Unmodified and sulfonated SF derivatives were evaluated by FTIR spectra. The peaks around 1658 cm^−1^ (amide I) and 1540 cm^−1^ (amide II) which are characteristic absorption bands for peptides and proteins, were shifted to 1625 cm^−1^ and 1520 cm^−1^, respectively, when treated with the diazonium coupling mixture. These findings indicate the transition from a random coil to a b-sheet structure, which are similar to the results observed in previous studies^28, 30, 31^. Hence, the sulfonated SF derivatives with different modification ratios can enhance β-sheet structure formation by exposure to methanol. Additionally, we detected a non-specific absorption band around 1380 cm^−1^ in the sulfonated groups, as a further proof to verify that the sulfonation process has occurred. (Fig. 1A). We then assessed the hydrophilicity of native SF and sulfonated SF derivatives by performing advancing contact angle measurement prior to chemical treatment (Fig. 1B). Films of native SF displayed contact angles of approximately 86°, whereas the contact angles of sulfonated SF films were gradually reduced with increasing levels of sulfonation. Both native SF and sulfonated SF derivatives could adhere onto the grid tightly. Scanning electron microscopy (SEM) showed that both the 3D printed gelatin grid coated by pure SF (3DG-SF, pore size = 100~200 μm) and the 3D printed gelatin grid coated by sulfonated SF (3DG-SF-SO_3_, pore size = 400~500 μm.) displayed porous structures (Fig. 1C). Higher magnification images showed that the former retained a relatively smooth surface, while the latter became rough and exhibited more porosity. Additionally, it was also observed that the sulfonated SF derivative scaffold formed a highly interconnected structure. The porosity of 3DG, 3DG-SF, 3DG-SF-SO_3_ were ~82.1%, ~88.0%, and ~87.6%, respectively. (SFig. 1).

### Effects of FGF-2 on cellular behavior

**Figure 2:**
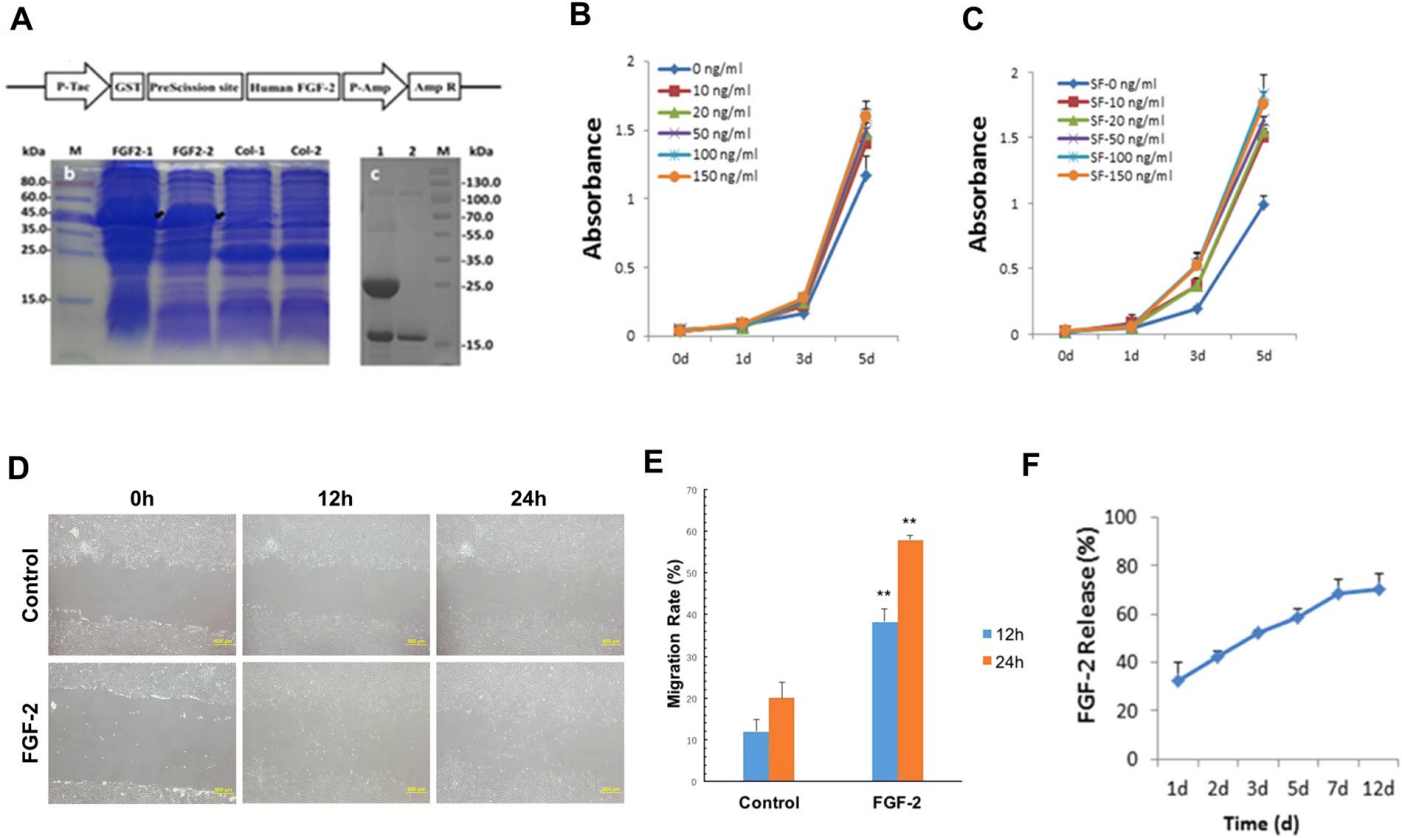
Effects of FGF-2 on child foreskin fibroblasts (CFFs). Purification of FGF-2 (**A**), and effects of free FGF-2 (**B**) and FGF-2 released from scaffold (**C**) on CFFs proliferation during 5-day culture time. Effects of free FGF-2 (100 ng/ml) on cell migration at 0 h, 12 h and 24 h, n = 3; Scale bars, 500 μm. (**D**–**E**) and FGF-2 (initial concentration:100 ng/ml) is continuously released from the scaffold over time, n = 3. (**F**).

FGF-2 was cloned from the cDNA of A549 cells (human lung adenocarcinoma cell line) and was subsequently expressed in *Escherichia coli* BL21 through the plasmid pGEX-6P-1 GST Expression Vector according to previous studies^32, 33^. Highly purified FGF-2 with a single band at ~17 kDa was obtained after protein purification (Fig. 2A). The biological effects of FGF-2 at different concentrations were evaluated on child foreskin fibroblasts (CFFs), including cell proliferation and migration. CCK-8 assays showed that cells exhibited similar proliferation rates with or without FGF-2 on both day 1 and day 3; however, on day 5, the enhancement of proliferation rate was observed to be the most significantly increased (approximately 40%) when treated with FGF-2 at 100 ng/ml concentration (Fig. 2B).

Effects of the released FGF-2 at different concentrations from the 3DG-SF-SO_3_-FGF scaffold on CFF proliferation were also evaluated. The results illustrated a similar pattern with a rate of 75% increase in proliferation rate at 100 ng/ml FGF-2, which is the optimal concentration (Fig. 2C). Hence, we utilized 100 ng/ml FGF-2 for further studies. Moreover, results of the wound healing assay demonstrated a significant enhancement of CFF migration with an addition of FGF-2 (100 ng/ml) (Fig. 2D,E). The *in vitro* release profile of FGF-2 from the 3DG-SF-SO_3_-FGF scaffold was demonstrated in Fig. 2F. A burst release of 32% was detected during the first initial 24 hours, followed by a constant slow-release profile. By day 12, 30% of the incorporated FGF-2 still remained within the scaffold. These data collectively suggest that both free and scaffold-adsorbed FGF-2 possess bioactivity, and that the latter format exhibited a sustained release profile.

### Morphology and proliferation of CFF on 3D printed scaffolds

**Figure 3:**
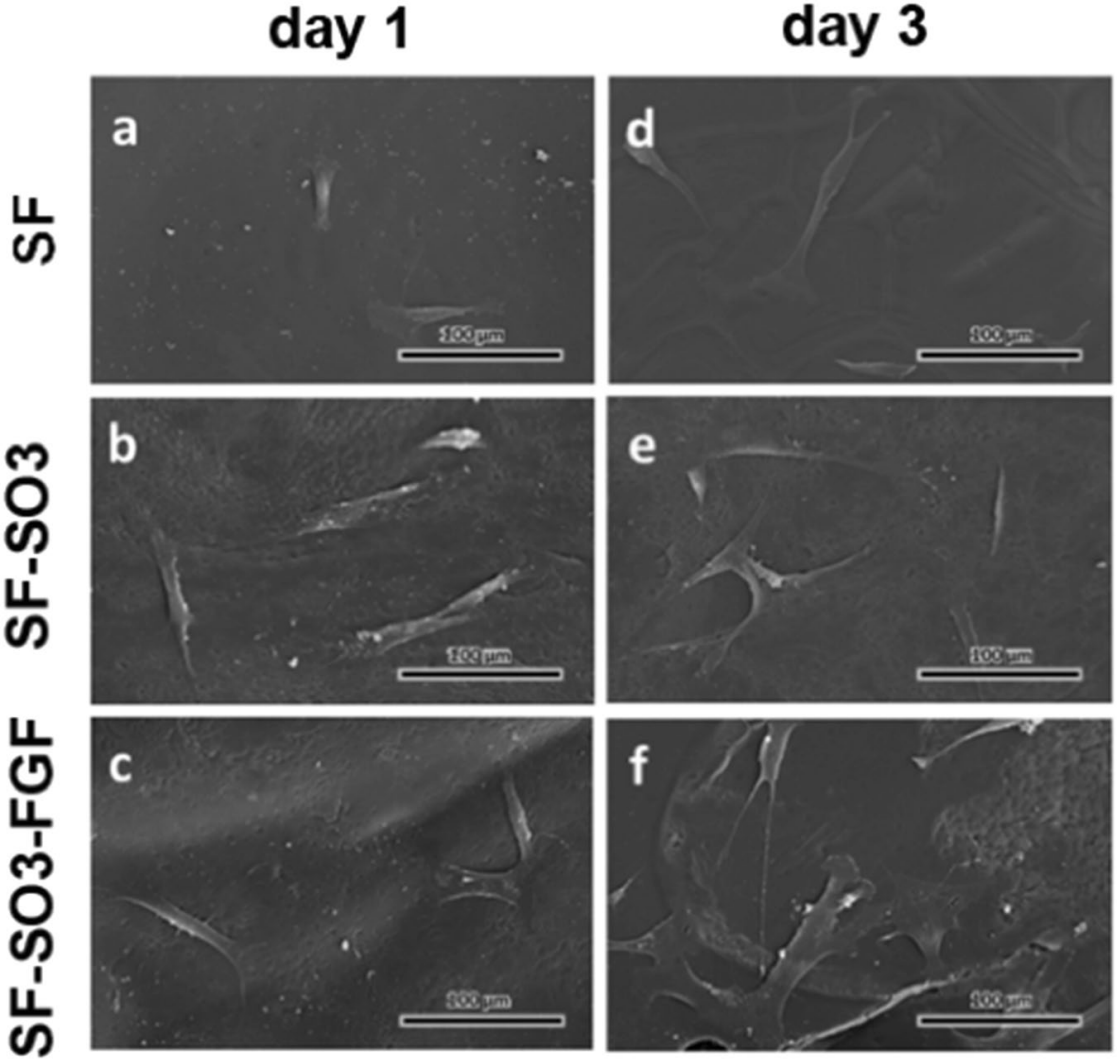
Morphology of CFFs cultured on pure SF scaffolds (**a** and **d**), sulfated SF (SF-SO_3_) scaffolds (**b** and **e**) and FGF-2 incorporated sulfonated SF (SF-SO_3_-FGF) scaffolds (**c** and **f**) at day 1 and day 3. Magnification = 400X. n = 3; Scale bars, 100 μm.

To assess the effects of the sulfonated SF derivative on cell adhesion and growth, we seeded CFFs on native SF scaffolds, sulfonated SF scaffolds and FGF-2 modified sulfonated SF scaffolds, and observed cell morphologies by SEM on day 1 and day 3 (Fig. 3). SEM micrographs indicated that CFFs can adhere to the native SF scaffold (Fig. 3a and d), sulfonated SF scaffold (Fig. 3b and e) and FGF-2 incorporated sulfonated SF scaffold (Fig. 3c and f). After 3 days in culture, the number of adherent cells was visualized to be higher on the FGF-2 incorporated sulfonated SF scaffolds, as compared to the native and sulfonated SF scaffold (Fig. 3f). Additionally, cells cultured on sulfonated SF scaffolds exhibited more spreading, covering greater cell attachment surface areas (Fig. 3e and f). Hence, these results conclusively demonstrated that sulfonated SF coated scaffolds support cell adhesion, spreading, and growth.

### Effects of 3D printed FGF-2 incorporated scaffolds on skin repair and blood vessel formation

**Figure 4:**
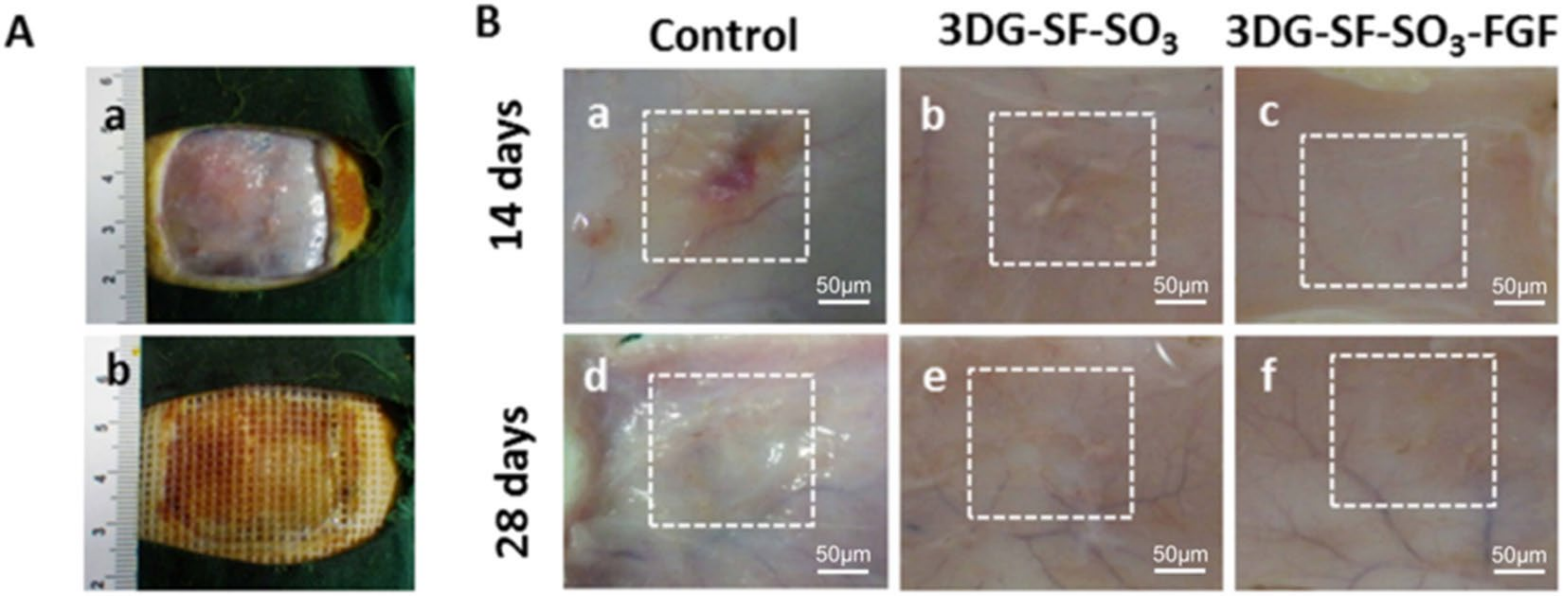
3DG-SF-SO_3_-FGF scaffolds were applied to full-thickness skin wounds and compared to wounds treated with petroleum gauze (control) or 3DG-SF-SO_3_ scaffolds. (**A**) Micrographs of animal surgery (a,b). (**B**) Gross observation of angiogenesis within newly formed tissues of healing wounds at day 14 and day 28 post-surgery. Scale bars, 50 μm.

In order to evaluate the effects of 3D printed scaffolds on skin repair, both 3DG-SF-SO_3_-FGF scaffolds and 3DG-SF-SO_3_ scaffolds were transplanted onto a full-thickness skin defect on the rat dorsal surface (Fig. 4A)^34, 35^. In order to observe wound healing and vascularization, newly formed tissues from the dorsal skin of the rat back were retrieved and visualized quickly after 14 and 28 days postoperatively. The results showed that the 3DG-SF-SO_3_-FGF scaffold helped smoothen the wounds after surgery, and more blood vessels were visible in the 3DG-SF-SO_3_-FGF group as shown in Fig. 4B(c and f).

**Figure 5:**
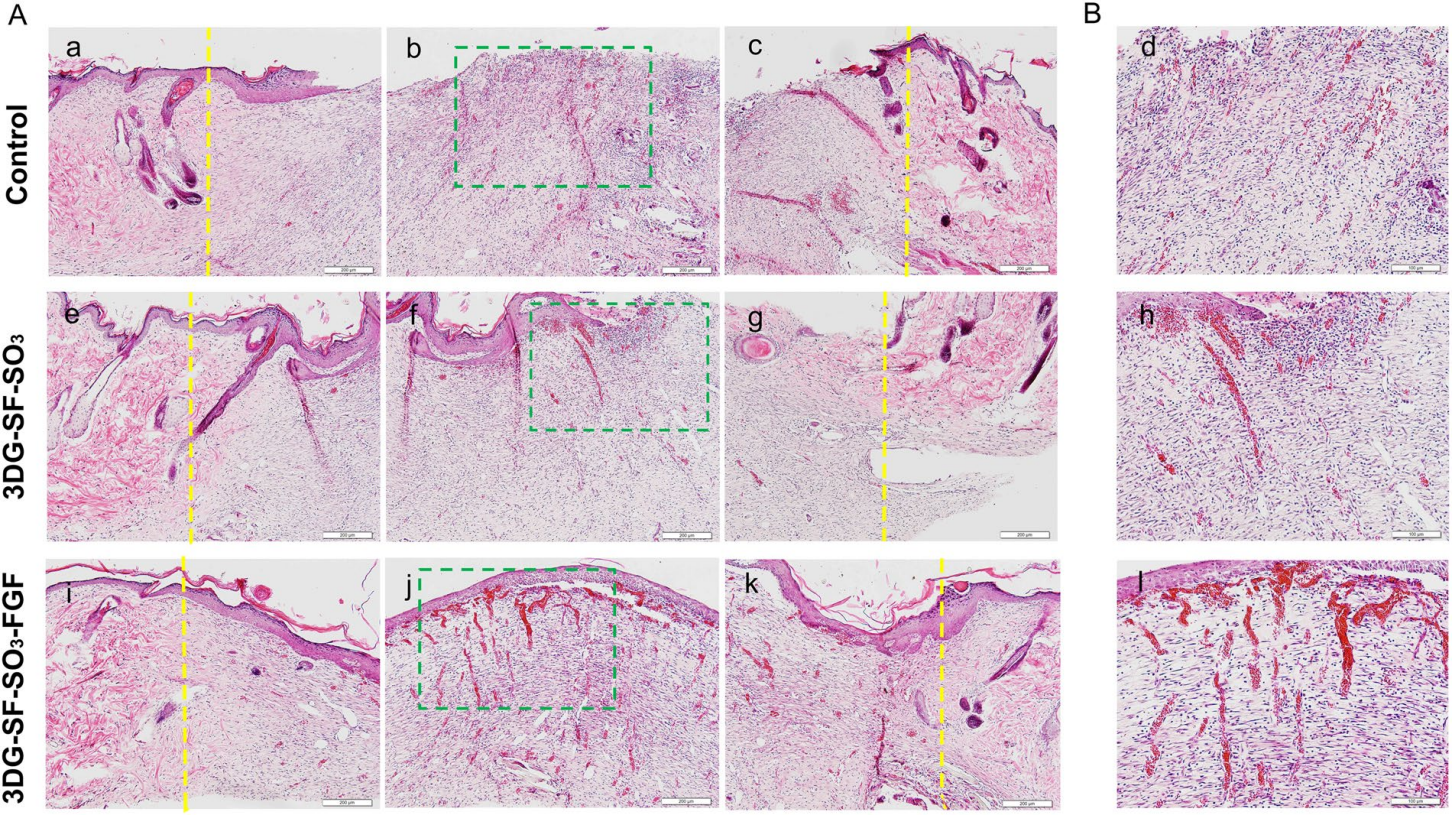
Representative H&E staining of skin tissue sections at 14 days post-surgery. which illustrate the healing process at the left (a,e,i), centre (b,f,j) and right regions of the wound (c,g,k). Yellow lines in the images indicated the boundaries between the wound and the surrounding normal skin. (**B**) Magnified view (d,h,l) of the regions in green pane in figure (**A**) (b,f and j). Scale bars = 200 μm (**A**), and 100 μm (**B**) respectively.

**Figure 6:**
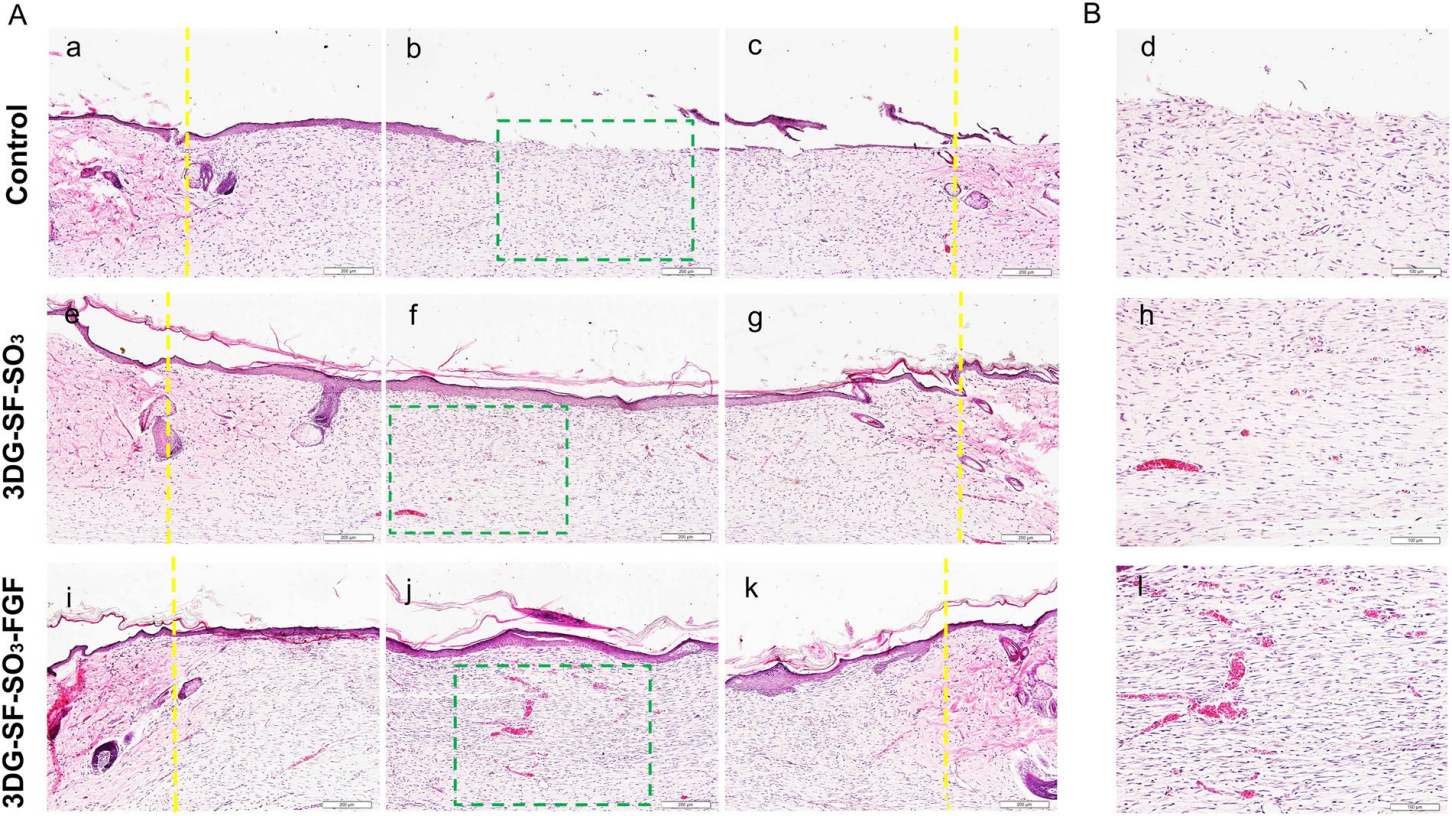
Representative H&E staining of skin tissue sections at day 28 post-surgery. Which illustrate the healing process at the left (a,e,i), centre (b,f,j), and right of the wound (c,g,k). Yellow lines in the images indicated the boundaries between the wound and the surrounding normal skin. (**B**) Magnified view (d,h,l) of the regions in green pane in figure (**A**) (b, f and j). Scale bars = 200 μm (**A**), and 100 μm (**B**) respectively.

Histological staining was also employed to evaluate wound healing on samples harvested on day 14 and day 28 post-implantation (Figs 5 and 6). As shown in Fig. 5, the three columns represent the left, middle and right regions of the wound. The yellow line indicates the boundary between the wound and the surrounding normal skin. In all three groups, fibroblasts migrated from the wound bed, and granulation was initiated as the defect site was replaced with regenerating tissues (Fig. 5A,B). Compared with the petroleum gauze group (Fig. 5a,b and c), where sparse and bare epithelial layers were formed at the wound site, epithelial cells displayed a tendency to migrate from the edge of normal skin towards the center of the wound in the 3DG-SF-SO_3_ group (Fig. 5e,f and g). In contrast, almost completely repaired dermis and epidermis layers were displayed in the 3DG-SF-SO_3_-FGF group on day 14 post-implantation (Fig. 5i,j and k). These differences were further confirmed by visualizing magnified images of the middle region of the wound (Fig. 5d,h and l). Additionally, within the magnified images, small vessels and fresh red blood cells can be detected in the dermal layer of the 3DG-SF-SO_3_ and 3DG-SF-SO_3_-FGF groups (Fig. 5h and l). The revascularization was observed to be more widely distributed in the FGF-2 incorporated scaffold group, as compared to the scaffold alone group. By contrast, revascularization can hardly be observed in the control group (Fig. 5d).

By day 28 post-surgery, the defect sites were observed to be completely closed by re-epithelization in both the 3D printed scaffold groups (3DG-SF-SO_3_ and 3DG-SF-SO_3_-FGF), while the epidermis layer was still not completely formed at the center of the wound in the control group (Fig. 6a–c,e–g,i–k). As shown in Fig. 6d,h and l, blood vessels were presented sporadically when treated with petroleum gauze. Upon comparing blood vessel formation, it was observed that the number of blood vessels presented on day 28 decreased dramatically when compared to day 14, and exhibited a more normal blood vessel morphology. (Fig. 6d,h,l).

**Figure 7:**
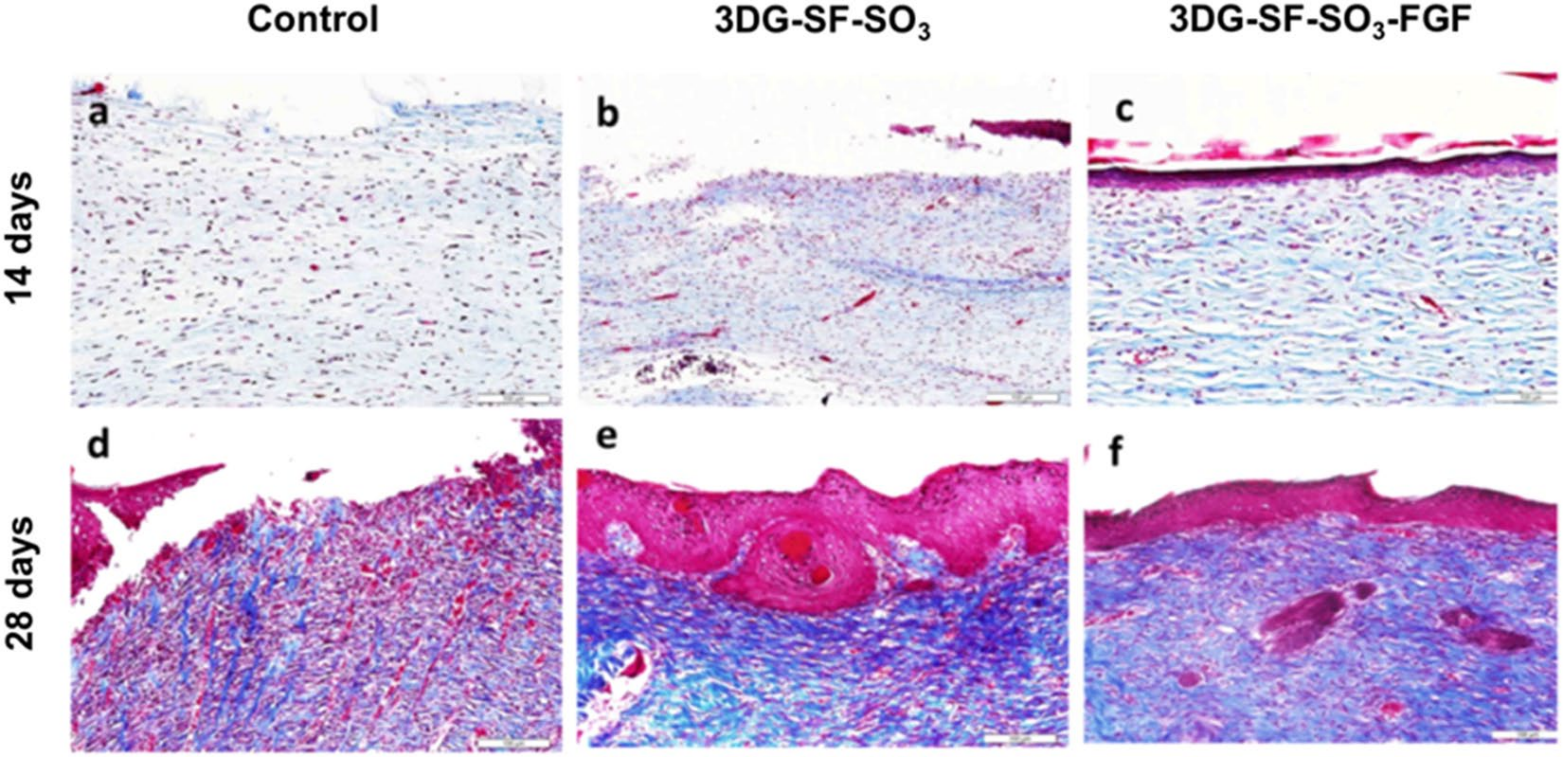
Masson trichrome staining shows histology of repaired wound after treatment. with petroleum gauze (**a**,**d**), 3DG-SF-SO_3_ scaffold (**b**,**e**) and 3DG-SF-SO_3_-FGF scaffold (**c**,**f**) at 14 and 28 days post-surgery. Scale bars = 100 μm.

Masson trichrome staining showed that the collagen content increased over time after implantation. On day 14 post-surgery, the histological appearance of skin defects implanted with the 3DG-SF-SO_3_-FGF scaffolds displayed organized collagen fibers with corrugated structure (Fig. 7c), whereas collagen fibers in the 3DG-SF-SO_3_ scaffolds (Fig. 7b) and control groups (Fig. 7a) were highly disordered. By day 28 post-surgery, collagen was more highly expressed in the 3DG-SF-SO_3_-FGF (Fig. 7f) and 3DG-SF-SO_3_ (Fig. 7e) groups, as compared to the control group (Fig. 7d).

### Blood Vessel and Epidermis Formation in Skin Defects

**Figure 8:**
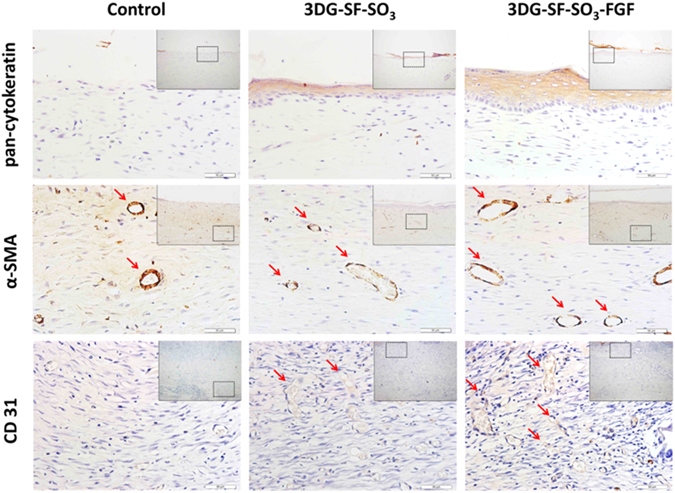
Epidermis and blood vessel formation in skin defects. Immunohistochemical staining of wound sections to detect expression of cytokeratin, SMA and CD31, after implantation with 3DG-SF-SO_3_ and 3DG-SF-SO_3_-FGF scaffolds at day 28 post-surgery. Scale bars = 50 μm.

For further characterization of blood vessel and epidermis formation in the skin defects, immunohistochemistry was performed to analyze expression levels of α-SMA and CD31 on day 28 post-surgery. As shown in the upper panel of Fig. 8, in contrast to the control group, pan-cytokeratin expression showed complete re-epithelialization in both 3D printed scaffolds. However, the staining results showed a significantly thicker epithelial layer in the 3DG-SF-SO_3_-FGF group when compared to scaffolds lacking FGF-2. Similarly, immunostaining for blood vessel related proteins α-SMA and CD31 showed that these were up-regulated upon the release of FGF-2 from the 3DG-SF-SO_3_-FGF scaffold, as compared to the control group.

## Discussion

In this study, we demonstrated that 3D printed gelatin-based silk fibroin (SF) scaffolds promoted healing of full-thickness skin defects, and that incorporation of FGF-2 could further enhance the treatment efficacy. Modified SF with sulfonated moieties was utilized in the scaffold to increase its hydrophilicity, as well as facilitate incorporation of FGF-2. It was demonstrated that the immobilized growth factor FGF-2 exhibited sustained release kinetics and was able to stimulate cell proliferation and migration *in vitro*. Subsequently, the 3D printed scaffold exhibited promising results when utilized to treat full-thickness cutaneous injuries in an animal model. As demonstrated in the results, incorporation of FGF-2 within the scaffold enhanced proliferation rate (from ~40% to ~75%), tissue morphology, collagen fibril assembly, blood vessel formation, and expression of various corresponding markers. These data thus suggest that recombinant FGF-2 delivered by the 3D printed scaffold could be a viable and innovative therapeutic strategy for severe skin defects.

Increasing evidence from previous studies suggest that silk composite scaffolds display positive efficacy in full-thickness skin regeneration and wound healing upon incorporation with specific growth factors and cells^36–38^. Although proven to be beneficial, the relatively slow degradation rate of silk composite scaffolds is incompatible with regenerating tissues, thus reducing its efficacy. In this study, we evaluated the degradation rate of pure silk fibroin in two different solutions: 0.1 M PBS buffer and 1 mg/ml alpha-chymotrypsin solution, and no significant degradation was observed in both solutions (SFig. 2). In contrast, the tensile strength were decreased to ~60% when SF scaffolds were immersed in alpha-chymotrypsin solution after 42 days (SFig. 3). Therefore, blending SF with other materials like gelatin is utilized in order to accelerate the degradation rate.

As a collagen derivative, gelatin possesses good biocompatibility and exhibits fast degradation rate, which is advantageous for tissue engineering applications. Currently, there are increasing numbers of studies on the use of gelatin and silk fibroin interpenetrating polymer composite scaffolds fabricated by various means (chemical cross-linking or simple blending) for diverse tissue engineering applications, with the scaffold functioning as either a structural component of tissue engineering or to facilitate drug delivery^39–41^. Previous studies have shown that gelatin-incorporated scaffolds were able to stimulate fibroblasts adhesion and proliferation. However, only a few studies have investigated this type of composite biopolymer scaffold for skin wound healing^42–44^.

In this study, a novel strategy was employed in which the basic structural framework was composed of fast-degrading gelatin, with its surface and the mesh being covered or filled with silk fibroin. By combining with SF and sulfonated SF, the pure gelatin scaffold was provided with enhanced mechanical support (SFig. 4). After confirming the relatively low cytotoxicity of the composite *in vitro* (SFig. 5), we further investigated its advantages of *in vivo* applications. A complex cascade of highly orchestrated and overlapping processes of clotting (hemostasis), re-epithelialization (migration and proliferation), and remodeling (maturation) naturally takes place after skin injuries. Wound granulation is an important stage in skin healing, and represents a critical part of the migration and proliferation phase^45^. Upon gelatin degradation, the SF mesh provides a microscale porous structure that subsequently facilitates wound granulation by guiding columnar tissue to grow through the mesh and fills up the wound in order to restore function^46^.

Generally, epithelialization can only take place after the granulated tissue reaches the level of the surrounding skin, while epithelial cells migrate across the new tissue to form a barrier between the wound and the environment^47^. However, as shown in Fig. 5 of our study, although granulation was observed in all three groups, no epithelialization occurred in the control group. In a recently proposed novel theoretical model, the re-epithelialization phase is driven by chemotaxis for a circular wound, and can be utilized to explain the observations in this study^48^. As a matter of fact, re-epithelialization is dependent on the synergistic effects of multiple factors, including physical microenvironment and growth factors.

Angiogenesis and neovascularization are crucial for skin wound healing, as newly formed blood vessels provide nutrition and oxygen to growing tissues^7^. FGF-2 is a multifunctional poly-peptide that promotes growth, migration and differentiation of a broad variety of cell types, including dermal fibroblasts, keratinocytes, endothelial cells and melanocytes^49^, which was demonstrated by the *in vitro* cell growth and scratch experiments. FGF-2 was also proved to stimulate VEGF expression, granulation tissue formation, and blood vessel maturation^50, 51^. In the *in vivo* skin wound repair model utilized in this study, immunohistochemistry displayed significantly increased expression of blood vessel related proteins such as α-SMA and CD31. Analysis of the FGF-2 release kinetics showed a continuous released of FGF-2 from the sulfonated silk scaffold, due to its binding with the sulfonated silk fibroin that mimicking the natural interactions between FGF-2 and the sulfated glycosaminoglycan heparan sulfate in the ECM. Thus, continuous release of FGF-2 from the scaffold contributed to the increased expression of blood vessel related proteins in the rat model. These data demonstrated the biological role of FGF-2 in recruiting endogenous cell for epithelialization and blood vessel formation at the defect site.

At present, techniques to incorporate growth factors within biomaterials mainly focuses on two strategies, direct mixing^52^ and chemical covalent linkage^53^. The major shortcomings of direct mixing are low loading efficiency and inadequate growth factor release. On the other hand, covalent linkage is associated with harsh chemical treatment that compromises the bioactivity of growth factors. In nature, association of growth factors and ECM in tissues involves non-covalent interactions i.e. electrostatic interactions. Modification of SF with sulfonic acid moieties could mimic the natural ECM and confer high capacity for sequestration, as well as enable sustained slow-release of FGF-2^31^. Hence, we utilized this non-covalent interaction between SF derivatives and FGF-2 to achieve a slow release system. Furthermore, addition of sulfonated moieties to SF can gradually increase the hydrophilicity of SF, which could result in enhanced cell adhesion and increased bioactivity on a more hydrophilic biomaterial surface. This may be the reason for the observed enhancement of cell adhesion in our study. The mechanical properties of silk depend mainly on its natural structure^54^. Although addition of chemical moieties to SF may alter the pore size, damage to the natural structure of silk fibroin was not observed (Fig. 1b and c).

Currently, cultured Epidermal Autograft (CEA) and Split Thickness Skin Graft (STSG) are the most widespread treatment modalities for severe burns in the clinic^55^. However, these grafts are not satisfactory and effective in repairing large area skin damage^56^ and chronic skin wounds due to lower granulation^57^, and lack of vascularization during the process of wound healing^35^. Our silk fibroin derivative has demonstrated great potential for such types of major cutaneous defects, and further investigations in big animal models will be performed in the near future.

## Materials and Methods

### Materials and chemical processing

*Bombyx mori* silk fibroin solution (6% w/v) was purchased from Zhejiang Cathaya International Inc. (Hangzhou, China). The diazonium coupling reaction was carried out as previously described^31^. Briefly, 4 mL of 4-sulfanilic acid (0.2 M) and 2 mL of aqueous solution of p-toluenesulfonic acid (1.6 M) were combined and quickly vortexed. Then, 2 mL of aqueous solution of NaNO_2_ (0.8 M) was added to the mixture before placing it on ice for 30 min. Subsequently, the SF solution was then reacted with the prepared salt solution in borate buffer. To control the level of sulfonation, different volumes of the diazonium salt (0, 1, 2, 3, or 4 volume) were added dropwise to the SF solution (16 volumes) in order to achieve the optimum composition. Sulfonated silk fibroin derivatives were purified by dialysis with distilled water for three days.

### Preparation of 3DG-SF-SO_3_-FGF scaffolds

Fabrication of the 3DG-SF-SO_3_ scaffold was comprised of three steps. Initially, gelatin powder was sterilized by UV-radiation and dissolved in distilled water at a concentration of 2% (w/v). A 2 cm * 2 cm gelatin grid (the basic network of the scaffold) was produced by a self-made pneumatic bio-printing system^58^ with a syringe nozzle of 100 μm and printing velocity of 5 mm/s within a laminar flow-cabinet. After printing, the scaffolds were cross-linked with 1% (m/v) EDC-NHS solution for 2 hrs. Then, the sulfonated silk fibroin derivative was poured onto the 3D printed scaffolds, followed by temporary storage at 4 °C for 4 hours and subsequent freezing (−80 °C) for 12 hrs. Finally, the scaffold was soaked in FGF-2 solution (100 μg/ml) overnight to absorb the growth factor, followed by storage after sterilization through gamma irradiation. The intensity used for sterilization is 20~25 KGy. Since the thickness of the epidermis and dermis layer is theoretically thought to be between 1–10 mm depending on the specific part of the body, we chose to print the gelatin scaffolds mimicking the skin thickness within the normal range.

### Scaffolds characterization

Films of native SF and sulfonated SF derivatives were characterized directly by Fourier transform infrared spectroscopy (Nicolet, AVA TAR370) as previously described. Aqueous solutions (6%) of native SF and sulfonated SF derivatives were spread on glass slides and dried overnight. Films were then exposed to water vapor of 96% relative humidity by equilibration in the presence of a saturated aqueous Na_2_SO_4_ solution at room temperature for 12 h, as previously described^59, 60^, followed by drying. This treatment is denoted as water vapor treatment. Advancing water-contact angles were analyzed using a drop shape analyzer. The porosities of 3D gelatin scaffold and 3D gelatin scaffold with native or sulfonated SF were tested by AutoPore IV 9500 V1.07 (Micromeritics Instrument Corporation).

### Mechanical evaluation

The mechanical stretch properties of the 3D gelatin scaffold and 3D gelatin scaffold with native or sulfonated SF were tested by using the Instron testing machine (model 5543; Instron, Canton, MA) and software (Bluehill V2.0; Instron). The unconfined equilibrium modulus was also determined by applying a step displacement (20% strain), monitoring the stress in relations with time until equilibrium was reached. Physical thickness of the scaffolds was measured, and the stress-strain relations were evaluated. The crosshead speed used was approximately 0.06 mm/min. The ratio of equilibrium force to cross-sectional area was divided by the applied strain to calculate the equilibrium modulus (in MPa).

### FGF-2 release from 3DG-SF-SO_3_-FGF scaffolds

To analyze the FGF-2 release profile from 3DG-SF-SO_3_-FGF scaffolds, the whole scaffold was soaked in PBS and the medium was collected at different time points, with 3DG-SF-SO_3_ scaffolds being represented as a control group. Protein concentrations of FGF-2 within the medium were then determined by a kit (Micro BCA Protein Assay Kit; Thermo Scientific).

### Effects of FGF-2 on cell proliferation

The child foreskin fibroblasts (CFFs) were kindly provided by Prof. Ji Junfeng, and CFFs were obtained from two donors, 10 aliquots from each donor. All cells were cultured in DMEM supplemented with 10% (v/v) fetal bovine serum at 37 °C within a humidified atmosphere of 5% CO_2_ and 95% air, and the medium was replaced every 3 days. Proliferation of child foreskin fibroblasts (CFF) was assayed by the Cell Counting KIT-8 (CCK-8) according to the manufacturer’s instructions. Cells were cultured on scaffolds in growth medium containing different concentrations of FGF-2 (0, 10, 20, 50, 100 and 150 ng/ml) for 1, 3 and 5 days, followed by incubation in 10% (v/v) CCK-8 solution in a 5% CO_2_ incubator at 37 °C for 3 h. The absorbance of the culture medium was then measured at 450 nm. Effects of the released FGF-2 from the 3DG-SF-SO_3_-FGF scaffold on CFF proliferation were also examined using the same method.

### Cell migration assay

CFF (1 × 10^5^) were seeded in 6-well culture plates to form a confluent monolayer. After incubation with FGF-2 for about 6 h, the cell monolayer was scraped in a straight line to create a “scratch” with a p200 pipet tip. The culture plate was then placed back into the incubator for 0 h, 12 h, 24 h, and digital images of the scratched region were captured under a phase-contrast microscopy. The rate of migration was determined by quantifying the average distance of the cellular movement from the edge towards the center of the scratch by Image Pro-Plus software^61^.

### Observation of cell morphology

CFF were cultured on 3D printed scaffolds at a density of 1 × 10^4^ cells/ml. Specimens were harvested on days 1 and 3 and fixed in 2.5% (w/v) glutaraldehyde solution for 24 h. After rinsing three times in PBS, the specimens were immersed in OsO_4_ (Ted Pella) for 1 h and then rinsed 3 times again in PBS. Subsequently, specimens were dehydrated in increasing concentrations of acetone, then mounted on aluminum stubs, coated with gold, and characterized by SEM (Hitachi S-3000N, Japan) at an accelerating voltage of 15 kV or 20 kV. For cell-free scaffolds, specimens were mounted on aluminum stubs and coated directly with gold.

### Cytotoxicity evaluation of scaffolds

The cytotoxicity of scaffolds was assessed according to the Biological Evaluation of Medical Devices protocol (GB/T 16886.5-2003/ISO 10993-5:1999). Three groups of medium were prepared. The basic medium consisted of high glucose DMEM with 10% (v/v) FBS, which was assigned as the blank control group. The positive control group was constituted of the basic medium with 5% (v/v) DMSO (Sigma–Aldrich Inc., St. Louis, USA). For the experimental group, sterilized scaffolds were soaked in 10 ml of the basic medium for more than 48 h in 37 °C before utilization. 100 ul aliquots of L929 cell suspension (1 × 10^4^/ml) (Chinese Academy of Sciences, Beijing, China) constituted in basic medium, were seeded in every single well of 96-well plates (Corning Inc., NY, USA),. Twenty-four hours later, the original medium was replaced with the basic medium, positive control group medium, and experimental group medium. L929 proliferation was assessed by the CCK-8 kit with absorbance readings being measured at 450 nm after 72 h in culture^62^.

### Rat skin defect model

All animal procedures complied with the Guidelines for the Care and Use of Laboratory Animals and were under the supervision of the Institutional Animal Care and Use Committee of Zhejiang University (ethics approval number: ZJU20160305). 36 male Sprague Dawley rats weighing 200–220 g were randomly assigned to two groups, and each group was further sub-divided into two time points (14 and 28 days post-surgery). After general anesthesia with 10% (w/v) chloralic hydras (400 mg/kg bodyweight), a full-thickness defect of 20 mm * 20 mm in dimensions extending through the panniculus carnosus was generated on the back of the rats^63^. The defects were implanted with sulfonated SF coated scaffolds (3DG-SF-SO_3_), sulfonated SF coated scaffolds with FGF-2 (3DG-SF-SO_3_-FGF), or petroleum gauze (control). Gauze pads were used to prevent removal of grafts from the wound. Post-operatively, animals were allowed to return to their cage after surgery.

### Gross morphology, Histological examination and Masson trichrome staining

At 14 and 28 days post-surgery, rats were sacrificed by an intravenous overdose of pentobarbital. Harvested specimens were immediately fixed in 4% (w/v) neutral buffered formalin for 24 h, then dehydrated through an alcohol gradient, and embedded in paraffin blocks. Histological sections (6 μm) were prepared using a microtome and were subsequently stained with hematoxylin and eosin (HE). Masson trichrome staining was performed on the basis of standard experimental procedures to compare the gross morphology of collagen fibers between the experimental and control groups^64^. The stained sections were photographed digitally under a microscope.

### Immunohistochemistry

To analyze epithelialization and vascularization of the regenerated tissues, immune-histochemical staining was performed on paraffin sections. Rat paraffin sections (6 µm in thickness) were incubated with 0.4% pepsin (Sangon Biotech, Shanghai, China) in 5 mM HCl at 37 °C for 20 min for antigen retrieval. Endogenous peroxidase was blocked by incubation with 3% (v/v) hydrogen peroxide in methanol for 5 min. Non-specific protein binding was blocked by incubation with 2% (w/v) BSA. After overnight incubation at 4 °C with primary antibodies, the sections were then incubated with goat anti-mouse (Beyotime Institute of Biotechnology Inc., Jiangsu, China) secondary antibodies for 2 h at room temperature. The DAB substrate system (Zsbio, Beijing, China) was used for color development. Finally, cell nuclei were stained with Hematoxylin and photographed digitally.

### Statistical and data analysis

All quantitative data were presented as mean ± standard deviation of at least 3 replicate samples from each group. Student’s t-test was performed to assess whether there were statistically significant differences in the results between groups. Significance levels were presented as either *p < 0.05 or **p < 0.01.

### Ethical approval

The animal experiments were under the supervision of the Institutional Animal Care and Use Committee of Zhejiang University (ethics approval number: ZJU20160305).

## Electronic supplementary material

Supplementary information

## References

[1] Blanpain C (2010). Stem cells: Skin regeneration and repair. Nature.

[2] Kaiser D, Hafner J, Mayer D, French LE, Lauchli S (2013). Alginate dressing and polyurethane film versus paraffin gauze in the treatment of split-thickness skin graft donor sites: a randomized controlled pilot study. Adv Skin Wound Care.

[3] Brem H (2007). Molecular markers in patients with chronic wounds to guide surgical debridement. Mol Med.

[4] Lin Q (2014). Pharmacological Mobilization of Endogenous Stem Cells Significantly Promotes Skin Regeneration after Full-Thickness Excision: The Synergistic Activity of AMD3100 and Tacrolimus. J Invest Dermatol.

[5] Filova E (2013). A cell-free nanofiber composite scaffold regenerated osteochondral defects in miniature pigs. Int J Pharm.

[6] Muylaert DE, Fledderus JO, Bouten CV, Dankers PY, Verhaar MC (2014). Combining tissue repair and tissue engineering; bioactivating implantable cell-free vascular scaffolds. Heart.

[7] Sun G (2011). Dextran hydrogel scaffolds enhance angiogenic responses and promote complete skin regeneration during burn wound healing. Proc Natl Acad Sci U S A.

[8] Murphy SV, Atala A (2014). 3D bioprinting of tissues and organs. Nat Biotechnol.

[9] Murphy SV, Skardal A, Atala A (2013). Evaluation of hydrogels for bio-printing applications. J Biomed Mater Res A.

[10] Lee V (2014). Design and fabrication of human skin by three-dimensional bioprinting. Tissue Eng Part C Methods.

[11] Pati F (2014). Printing three-dimensional tissue analogues with decellularized extracellular matrix bioink. Nat Commun.

[12] Xing Q (2014). Increasing mechanical strength of gelatin hydrogels by divalent metal ion removal. Sci Rep.

[13] Camci-Unal G, Cuttica D, Annabi N, Demarchi D, Khademhosseini A (2013). Synthesis and characterization of hybrid hyaluronic acid-gelatin hydrogels. Biomacromolecules.

[14] Wang Y, Kim HJ, Vunjak-Novakovic G, Kaplan DL (2006). Stem cell-based tissue engineering with silk biomaterials. Biomaterials.

[15] Jiang CY (2007). Mechanical properties of robust ultrathin silk fibroin films. Adv Funct Mater.

[16] Chen X (2008). Ligament regeneration using a knitted silk scaffold combined with collagen matrix. Biomaterials.

[17] Shen WL (2010). The effect of incorporation of exogenous stromal cell-derived factor-1 alpha within a knitted silk-collagen sponge scaffold on tendon regeneration. Biomaterials.

[18] Wang XW, Gu Y, Xiong ZP, Cui Z, Zhang T (2014). Silk-Molded Flexible, Ultrasensitive, and Highly Stable Electronic Skin for Monitoring Human Physiological Signals. Adv Mater.

[19] Cohen-Karni T (2012). Nanocomposite Gold-Silk Nanofibers. Nano Lett.

[20] Kasoju N, Bora U (2012). Silk Fibroin in Tissue Engineering. Adv Healthc Mater.

[21] Rockwood DN (2011). Materials fabrication from Bombyx mori silk fibroin. Nat Protoc.

[22] Seib FP, Pritchard EM, Kaplan DL (2013). Self-Assembling Doxorubicin Silk Hydrogels for the Focal Treatment of Primary Breast Cancer. Adv Funct Mater.

[23] Pritchard EM, Valentin T, Panilaitis B, Omenetto F, Kaplan DL (2013). Antibiotic-Releasing Silk Biomaterials for Infection Prevention and Treatment. Adv Funct Mater.

[24] Zhang XL (2013). Hierarchical biomineralization of calcium carbonate regulated by silk microspheres. Acta Biomater.

[25] Raja WK (2013). Transdermal Delivery Devices: Fabrication, Mechanics and Drug Release from Silk. Small.

[26] Wang Y (2008). *In vivo* degradation of three-dimensional silk fibroin scaffolds. Biomaterials.

[27] Numata K, Cebe P, Kaplan DL (2010). Mechanism of enzymatic degradation of beta-sheet crystals. Biomaterials.

[28] Jin HJ (2005). Water-stable silk films with reduced beta-sheet content. Adv Funct Mater.

[29] Yang Z (2012). *In vitro* and *in vivo* characterization of silk fibroin/gelatin composite scaffolds for liver tissue engineering. J Digest Dis.

[30] Li MZ, Ogiso M, Minoura N (2003). Enzymatic degradation behavior of porous silk fibroin sheets. Biomaterials.

[31] Murphy AR, John PS, Kaplan DL (2008). Modification of silk fibroin using diazonium coupling chemistry and the effects on hMSC proliferation and differentiation (vol 29, pg 2829, 2008). Biomaterials.

[32] Deng HX (2010). Prokaryotic expression, purification and characterization of a novel pro-apoptosis protein hPNAS-4. Biotechnol Appl Bioc.

[33] Song JA (2013). Expression and Purification of Biologically Active Human FGF2 Containing the b ‘ a ‘ Domains of Human PDI in Escherichia coli. Appl Biochem Biotech.

[34] Zhang ZY (2011). The role of single cell derived vascular resident endothelial progenitor cells in the enhancement of vascularization in scaffold-based skin regeneration. Biomaterials.

[35] Liu X (2013). RNAi functionalized collagen-chitosan/silicone membrane bilayer dermal equivalent for full-thickness skin regeneration with inhibited scarring. Biomaterials.

[36] Chouhan D, Chakraborty B, Nandi SK, Mandal BB (2017). Role of non-mulberry silk fibroin in deposition and regulation of extracellular matrix towards accelerated wound healing. Acta Biomater.

[37] Xie SY (2016). Adult Stem Cells Seeded on Electrospinning Silk Fibroin Nanofiberous Scaffold Enhance Wound Repair and Regeneration. J Nanosci Nanotechno.

[38] Min BM (2004). Electrospinning of silk fibroin nanofibers and its effect on the adhesion and spreading of normal human keratinocytes and fibroblasts *in vitro*. Biomaterials.

[39] Xiao WQ (2011). Synthesis and characterization of photocrosslinkable gelatin and silk fibroin interpenetrating polymer network hydrogels. Acta Biomater.

[40] Taddei P (2013). Silk Fibroin/Gelatin Blend Films Crosslinked with Enzymes for Biomedical Applications. Macromol Biosci.

[41] Dyakonov T (2012). Design and characterization of a silk-fibroin-based drug delivery platform using naproxen as a model drug. J Drug Deliv.

[42] Orlova AA, Kotlyarova MS, Lavrenov VS, Volkova SV, Arkhipova AY (2014). Relationship between Gelatin Concentrations in Silk Fibroin-Based Composite Scaffolds and Adhesion and Proliferation of Mouse Embryo Fibroblasts. B Exp Biol Med+.

[43] Kanokpanont S, Damrongsakkul S, Ratanavaraporn J, Aramwit P (2012). An innovative bi-layered wound dressing made of silk and gelatin for accelerated wound healing. Int J Pharmaceut.

[44] Lan Y (2014). Therapeutic efficacy of antibiotic-loaded gelatin microsphere/silk fibroin scaffolds in infected full-thickness burns. Acta Biomater.

[45] Pereira RF, Barrias CC, Granja PL, Bartolo PJ (2013). Advanced biofabrication strategies for skin regeneration and repair. Nanomedicine-Uk.

[46] Dearden C (2001). Traumatic wounds: the management of superficial and partial thickness burns. Nurs Times.

[47] Trexler RA (2011). Assessment of surgical wounds in the home health patient: definitions and accuracy with OASIS-C. Home Healthc Nurse.

[48] Amar, M. B. & Wu, M. Re-epithelialization: advancing epithelium frontier during wound healing. *J R Soc Interface* **11**, doi:ARTN 20131038.10.1098/rsif.2013.1038 (2014). 10.1098/rsif.2013.1038 PMC3928935 24451391

[49] Tiede S (2009). Basic fibroblast growth factor: A potential new therapeutic tool for the treatment of hypertrophic and keloid scars. Ann Anat.

[50] Wu J (2016). Heparin-Based Coacervate of FGF2 Improves Dermal Regeneration by Asserting a Synergistic Role with Cell Proliferation and Endogenous Facilitated VEGF for Cutaneous Wound Healing. Biomacromolecules.

[51] Claffey KP (2001). Fibroblast growth factor 2 activation of stromal cell vascular endothelial growth factor expression and angiogenesis. Lab Invest.

[52] Poldervaart, M. T. *et al*. Sustained Release of BMP-2 in Bioprinted Alginate for Osteogenicity in Mice and Rats. *Plos One* **8**, doi:ARTN e72610.10.1371/journal.pone.0072610 (2013). 10.1371/journal.pone.0072610 PMC3747086 23977328

[53] Chen PR, Chen MH, Lin FH, Su WY (2005). Release characteristics and bioactivity of gelatin-tricalcium phosphate membranes covalently immobilized with nerve growth factors. Biomaterials.

[54] Chen FJ, Porter D, Vollrath F (2012). Silk cocoon (Bombyx mori): Multi-layer structure and mechanical properties. Acta Biomater.

[55] Bottcher-Haberzeth S, Biedermann T, Reichmann E (2010). Tissue engineering of skin. Burns.

[56] Parmaksiz M, Elcin AE, Elcin YM (2015). Decellularization of bovine small intestinal submucosa and its use for the healing of a critical-sized full-thickness skin defect, alone and in combination with stem cells, in a small rodent model. J Tissue Eng Regen Med.

[57] Orgill DP (1998). Vascularized collagen-glycosaminoglycan matrix provides a dermal substrate and improves take of cultured epithelial autografts. Plast Reconstr Surg.

[58] Wang Q (2015). 3D-Printed Atsttrin-Incorporated Alginate/Hydroxyapatite Scaffold Promotes Bone Defect Regeneration with TNF/TNFR Signaling Involvement. Adv Healthc Mater.

[59] Wenk E, Wandrey AJ, Merkle HP, Meinel L (2008). Silk fibroin spheres as a platform for controlled drug delivery. J Control Release.

[60] Hino T, Tanimoto M, Shimabayashi S (2003). Change in secondary structure of silk fibroin during preparation of its microspheres by spray-drying and exposure to humid atmosphere. J Colloid Interf Sci.

[61] Liang CC, Park AY, Guan JL (2007). *In vitro* scratch assay: a convenient and inexpensive method for analysis of cell migration *in vitro*. Nat Protoc.

[62] Shi LB (2014). Tissue engineered bulking agent with adipose-derived stem cells and silk fibroin microspheres for the treatment of intrinsic urethral sphincter deficiency. Biomaterials.

[63] Ma B, Xie JW, Jiang J, Wu J (2014). Sandwich-type fiber scaffolds with square arrayed microwells and nanostructured cues as microskin grafts for skin regeneration. Biomaterials.

[64] Woo SLY, Takakura Y, Liang R, Jia FY, Moon DK (2006). Treatment with bioscaffold enhances the the fibril morphology and the collagen composition of healing medial collateral ligament in rabbits. Tissue Eng.

