# Supplementary material for: A Gelatin-sulfonated Silk Composite Scaffold based on 3D Printing Technology Enhances Skin Regeneration by Stimulating Epidermal Growth and Dermal Neovascularization
Source: Sci Rep. 2017 Jun 27;7:4288. doi: 10.1038/s41598-017-04149-y (PMC5487355; doi:10.1038/s41598-017-04149-y)
Supplement: Supplementary file 1 — Supplementary information [file 41598_2017_4149_MOESM1_ESM.pdf]

A Gelatin-sulfonated Silk Composite Scaffold based on 3D Printing Technology Enhances Skin Regeneration by Stimulating Epidermal Growth and Dermal Neovascularization

Si Xiong [a,b,f 1], Xianzhu Zhang [a,b,f 1], Ping Lu [a,b,f], Yan Wu [a,b,f], Quan Wang [a,b,f], Heng Sun [a,b,f], Boon Chin Heng [c], Varitsara Bunpetch [a,b,f], Shufang Zhang [a,b,f *], Hong-Wei Ouyang [a,b,d,e,f]

[a] Center for Stem Cell and Tissue Engineering, School of Medicine, Zhejiang University, China

[b] Zhejiang Provincial Key Laboratory of Tissue Engineering and Regenerative Medicine, Hangzhou, China

[c] University of Hong Kong, Faculty of Dentistry, Pokfulam, Hong Kong SAR, China

[d] State Key Laboratory for Diagnosis and Treatment of Infectious Diseases, Collaborative Innovation Center for Diagnosis and Treatment of Infectious Diseases , The First Affiliated Hospital, College of Medicine, Zhejiang University, Hangzhou, China

[e] Department of Sports Medicine, School of Medicine, Zhejiang university

[f] Dr. Li Dak Sum & Yip Yio Chin Center for Stem Cell and Regenerative Medicine, School of Medicine, Zhejiang University

Corresponding author.
Center for Stem Cell and Tissue Engineering, School of Medicine, Zhejiang University, Hangzhou, Zhejiang, China
Tel: +86-571-88208442; Fax: +86-571-88208262
Email address: zhangshufang@zju.edu.cn (S. Zhang)

Author Contributions

[1] The two authors contribute equally to this work. The manuscript was written through contributions of all authors. All authors have given approval to the final version of the manuscript.

Supplementary Figures

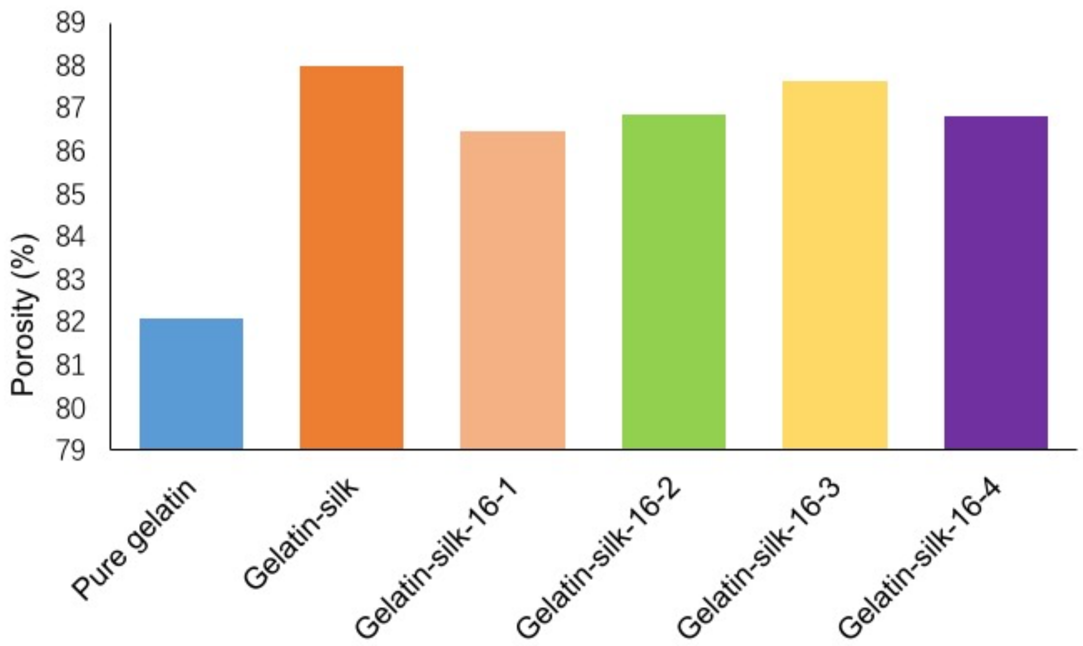

Supplementary Figure 1. The porosity (%) of 3D printing scaffolds with or without SF and sulfonated SF. Pure gelatin: Porosity=82.0799%, Median Pore Diameter (Volume) =
Gelatin-silk: Porosity=87.9883%, Median Pore Diameter (Volume)=99359.3nm. Gelatin-silk-16-1: Porosity=86.4762%, Median Pore Diameter (Volume)=110277.3nm. Gelatin-silk-16-2, Porosity=86.8367%, Median Pore Diameter (Volume)=123673.6nm. Gelatin-silk-16-3: Porosity=87.6422%, Median Pore Diameter (Volume)=170732.1nm. Gelatin-silk-16-4: Porosity=86.8103%, Median Pore Diameter (Volume) = 64888.5nm. Pure gelatin: 3D printing gelatin scaffold; Gelatin-silk: 3D printing gelatin scaffold covered with silk fibroin; Gelatin-silk-16-1: 3D printing gelatin scaffold covered with sulfonated silk fibroin (volume ratio of the SF solution to the diazonium salt, 16:1); Gelatin-silk-16-2: 3D printing gelatin scaffold covered with sulfonated silk fibroin (volume ratio of the SF solution to the diazonium salt, 16:2); Gelatin-silk-16-3: 3D printing gelatin scaffold covered with sulfonated silk fibroin (volume ratio of the SF solution to the diazonium salt, 16:3); Gelatin-silk-16-4: 3D printing gelatin scaffold covered with sulfonated silk fibroin (volume ratio of the SF solution to the diazonium salt, 16:4).

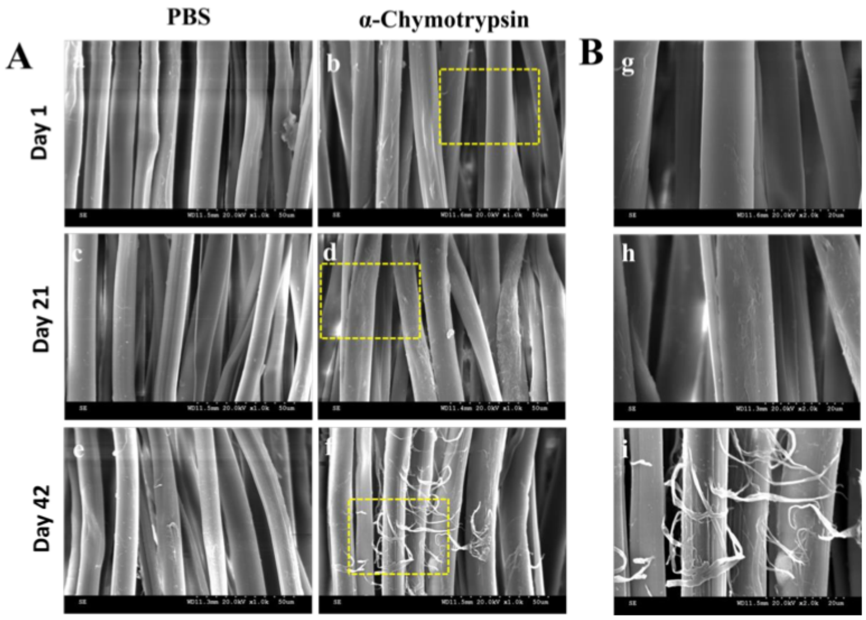

Supplementary Figure 2. The degradation of pure silk fibroin mesh observed by SEM in different solutions for 1, 21, and 42days. A-a,c,e. Pure silk fibroin mesh was degraded in 0.1M PBS buffer for day1, day21, and day42, respectively. Scale bar=50μm. A-b,d,f. Pure silk fibroin mesh was degraded in 1mg/ml alpha-chymotrypsin solution for day1, day21, and day42, respectively. Scale bar=50μm. B-g,h,i. High magnification in the yellow frame area in A-b,d,f. Scale bar=20μm.

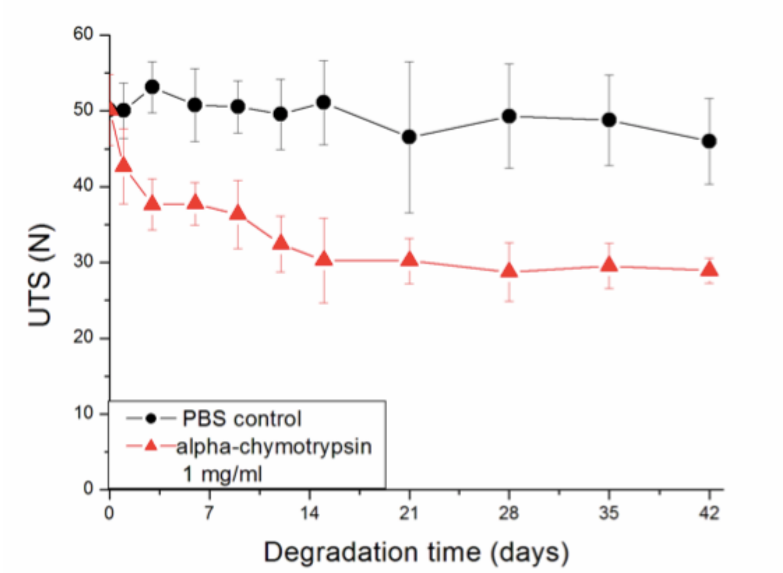

Supplementary Figure 3. Ultimate tensile strength of silk fibroin mesh after degradation in PBS solution and α–Chymotrypsin solution at different time points. The ultimate tensile strength of the silk fibroin mesh incubated in α-chymotrypsin degrading solution decreased gradually with the degradation time, and the mechanical strength decreased about 40%.

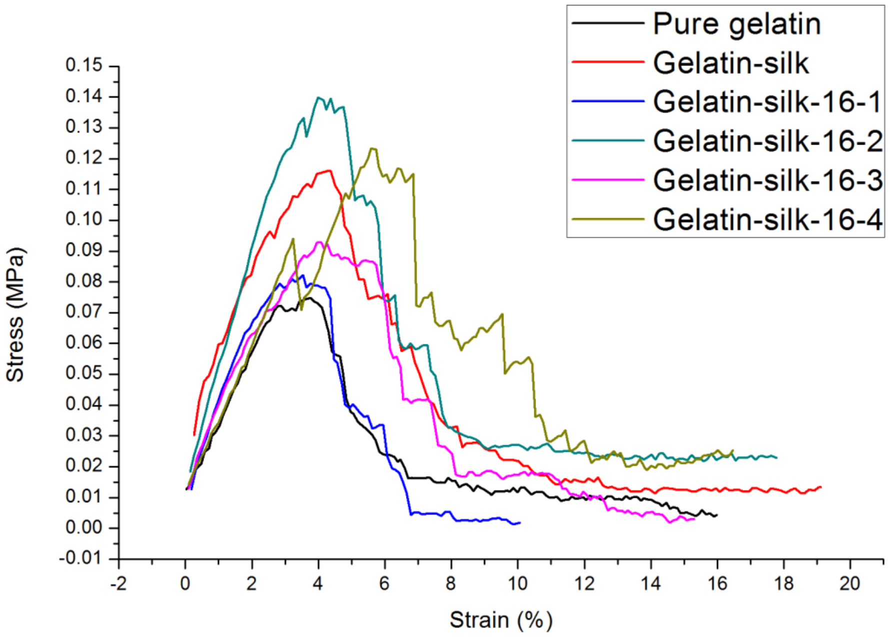

Supplementary Figure 4. Mechanical properties of 3D printing scaffolds with or without SF and sulfonated SF. Pure gelatin, 3D printing gelatin scaffold. Gelatin-silk, 3D printing gelatin scaffold covered with silk fibroin. Gelatin-silk-16-1, 3D printing gelatin scaffold covered with sulfonated silk fibroin (volume ratio of the SF solution to the diazonium salt, 16:1). Gelatin-silk-16-2, 16:2. Gelatin-silk-16-3, 16:3. Gelatin-silk-16-4, 16:4.

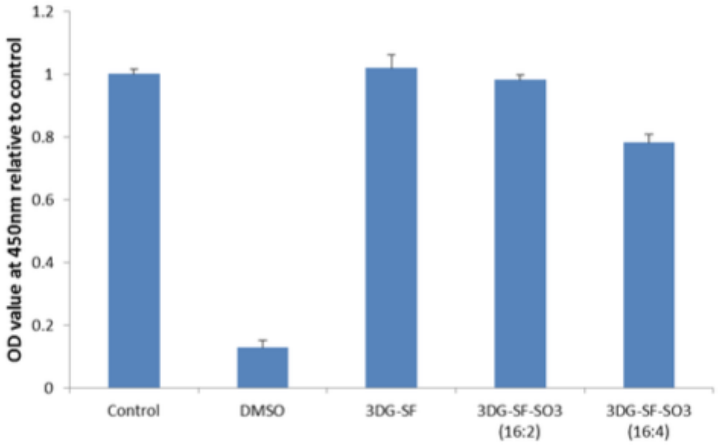

Supplementary Figure 5. Cytotoxicity of 3DG-SF and 3DG-SF-SO3 scaffold by CCK-8 test. The cytotoxicity of sulfonated silk fibroin (3DG-SF-SO3) at the ratio of 16: 2 (Silk fibroin solution to sulfonation reagent) belongs to level 1 (qualified), according to the Biological test program for medical devices - Cytotoxicity test (GB/T 16886.5). The cytotoxicity of sulfonated silk fibroin (3DG-SF-SO3) at the ratio of 16: 4 belongs to level 2 (qualified), which was also in line with the safety requirements for animal experiments.
